# Supplementary material for: Endophilin, Lamellipodin, and Mena cooperate to regulate F-actin-dependent EGF-receptor endocytosis
Source: EMBO J. 2013 Sep 27;32(20):2722–34. doi: 10.1038/emboj.2013.212 (PMC3801443; doi:10.1038/emboj.2013.212)
Supplement: Supplementary Information [file emboj2013212s1.pdf]

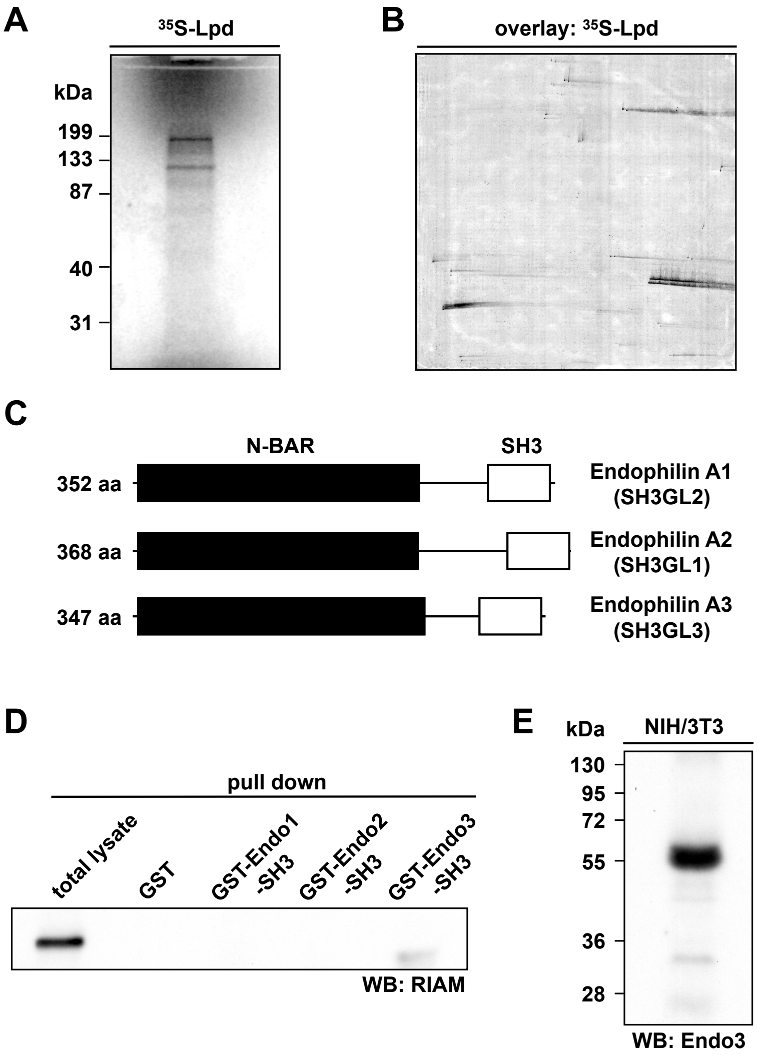

Supplemental Figure 1, Vehlow et al.

Figure S1: Identification of endophilin as a new Lamellipodin-interacting protein

(A) Autoradiography of ^{35}S-labelled Lpd. **(B)** Proteomic screen of a human fetal brain protein array with *in vitro* translated, ^{35}S-labeled full length Lpd as the bait. Dark double spots show positive hits. **(C)** Domain structure of proteins of the endophilin A family. **(D)** Pulldown of RIAM from NIH/3T3 cell lysate using GST-tagged SH3 domains of endophilin A1, endophilin A2 and endophilin A3 or GST as control. A representative blot from 3 independent experiments is shown. **(E)** Detection of endogenous endophilin A3 using specific antibodies in NIH/3T3 cell lysate.

A

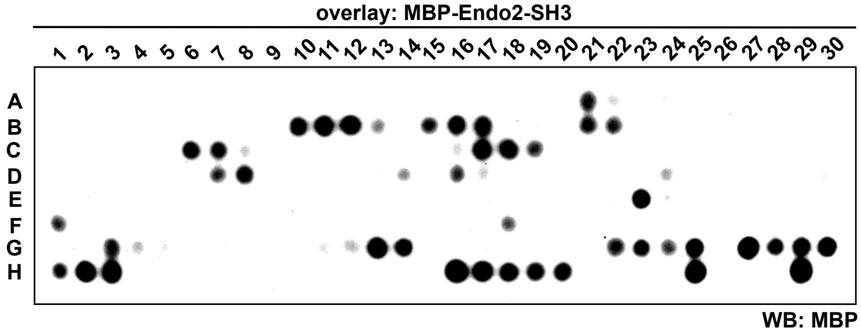

B

Lpd fragment	peptide	position		SH3 domain-binding motif
GST-Lpd-C1	SLVPPLSPQPKI	A21	+	PPxxPxPK
	APMFVKYSTITR	B10	++	false positive? (highly charged)
	FVKYSTITRLQN	B11	++	
	YSTITRLQNASQ	B12	++	
	ASQHSGALFKPP	B15	+	KPPxPP, KxPxxP
	HSGALFKPPTPP	B16	++	
	ALFKPPTPPVMQ	B17	++	
	SQSVKPQILVPP	B21	+	KPxxxxPP
	VKPQILVPPNGV	B22	+	
GST-Lpd-C2	QFSAPPPPLKIH	C6	++	PPPPxK, PxxPxK
	APPPPLKIHQVQ	C7	++	
	PLPPQAPPKPLV	C17	++	PxxPPKP, PxxPxK
	PQAPPKPLVTIP	C18	++	
	PPKPLVTIPAPT	C19	(+)	
GST-Lpd-C3	PVPVPPPTLPKQ	D7	(+)	PPPxxPK
	VPPPTLPKQQSF	D8	++	
	PSVVKQIASQFP	D16	(+)	
GST-Lpd-C4	KPPPTPQRNSSI	E23	++	KPPPxPxR, KxPxxP, PxxPxR
	RPSVDSLVSKFT	F1	(+)	
	CVSAKAPVLSGR	F18	(+)	
GST-Lpd-C5	PAVFSGNTSPKV	G3	+	
	PPPTRPKRNDST	G13	++	PPPxRPKR
	TRPKRNDSTRLT	G14	+	
GST-Lpd-C6	VVPQVPTSPKSS	G22	+	
	QVPTSPKSSLSV	G23	+	PxxPK
	TSPKSSLSVQPG	G24	(+)	
	KSSLSVQPGFLA	G25	+	
	QPGFLADLNRTL	G27	++	false positives?
	FLADLNRTLQRK	G28	+	false positive? (highly charged)
	DLNRTLQRKSIT	G29	++	false positive? (G27)
	RTLQRKSITRHG	G30	++	interaction of MBP with GST-Lpd-C6 (Fig. S2d)
	QRKSITRHGSLS	H1	(+)	
	SITRHGSLSSRM	H2	++	
	RHGSLSSRMSRA	H3	++	
	GGSHISGYATLR	H16	++	
	HISGYATLRRGP	H17	++	
	GYATLRRGPPPA	H18	+	
	TLRRGPPPAPPK	H19	+	RxPPPxPPK
	RGPPPAPPKRDQ	H20	+	
positve control	PPPPRPVAPPTR	H25	++	Endophilin A1-binding site in Synaptojanin
	PPPPRPVAPPTR	H29	++	
negative control	PPPALPSSAPSG	H23	-	
	PPPHSSGPPPPP	H24	-	
	PQAAFPPQSSLP	H26	-	
	PPPALPSSAPSG	H27	-	
	PPPHSSGPPPPP	H28	-	
	PQAAFPPQSSLP	H30	-	

Supplemental Figure 2, Vehlow et al.

Figure S2: Identification of endophilin SH3 domain binding sites within Lamellipodin

(A) SPOTS scan peptide array overlayed with MBP-Endo2-SH3. **(B)** Table showing SH3 domain binding motifs in the Lpd sequence identified in the SPOTS scan peptide array in **(A)**.

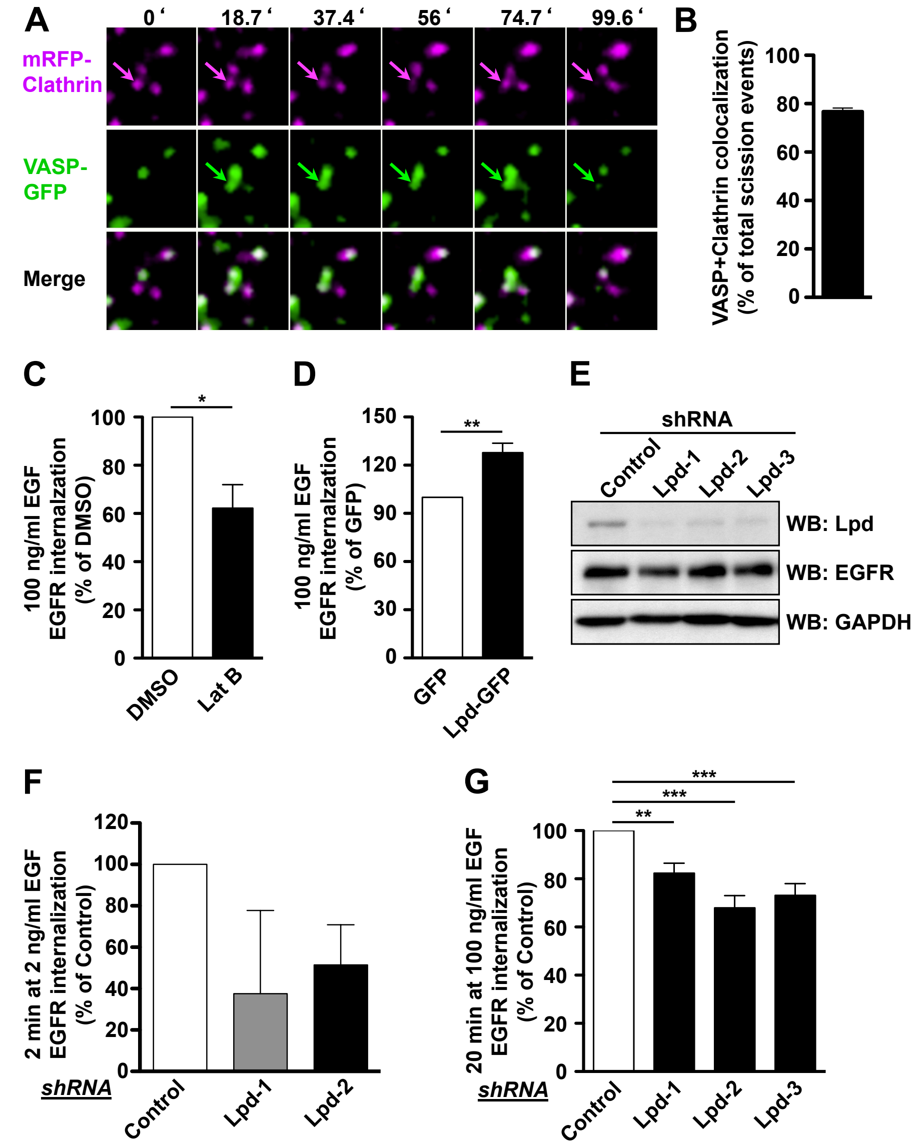

Supplemental Figure 3, Vehlow et al.

Figure S3: Lpd localizes to CCPs and regulates endocytosis of the EGFR

(A-B) Dynamics of VASP-GFP and mRFP-Clc in HeLa cells was assessed every 5 sec using TIRFM. Single color and merged images of an area of a representative cell are shown. Arrows show recruitment of VASP-GFP to mRFP-Clc shortly before scission. Scale bar: 1 μm. (See also Movie S2.) **(B)** Quantification of the percentage of scission events of CCPs containing mRFP-Clc and VASP-GFP. In total 361 scission events of 3 different cells were analyzed for each experiment. **(C)** EGFR internalization in HeLa cells treated with Latrunculin B (Lat B) or DMSO control and 100 ng/ml EGF. **(D)** EGFR internalization in HeLa overexpressing Lpd-GFP or GFP as control and treated with 100 ng/ml EGF. **(E)** Detection of endogenous Lpd and EGFR using specific antibodies in lysate of HeLa cells expressing three Lpd-specific or control shRNA. GAPDH serves as loading control. **(F-G)** EGFR internalization in HeLa cells expressing Lpd-specific or control shRNA and treated with 2 ng/ml **(F)** or 100 ng/ml **(G)** EGF for indicated times. **(C,D,F,G)** Results are mean ± SEM of at least 3 independent experiments. **(C-D)** t-test: *$p<0.05$, **$p<0.01$. **(G)** One-way ANOVA, Tukey's: *$p<0.05$, **$p<0.01$, ***$p<0.001$.

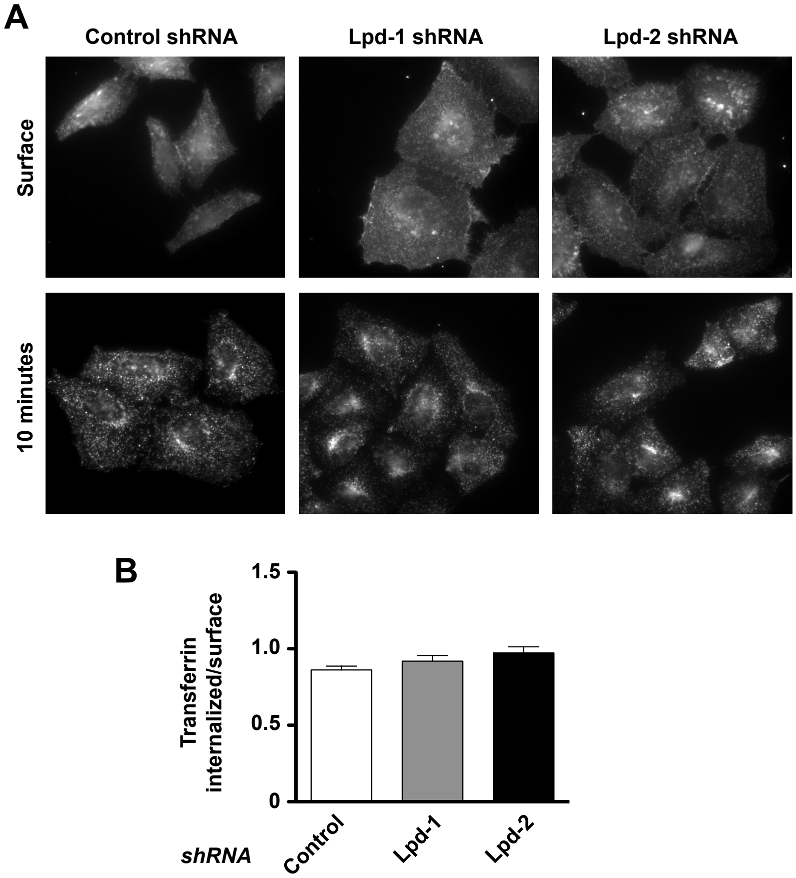

Supplemental Figure 4, Vehlow et al.

Figure S4: Lpd does not regulate constitutive transferrin receptor endocytosis.

(A,B) Transferrin uptake was quantified with an imaging based uptake assay. **(A)** Alexa-488 transferrin at the surface and internalized after 10 min incubation in control shRNA expressing and Lpd knockdown HeLa cells. **(B)** Transferrin uptake was quantified as intensity per area from 30 cells from 3 independent experiments. The ratio between the internalized and surface bound A488-transferrin is shown.

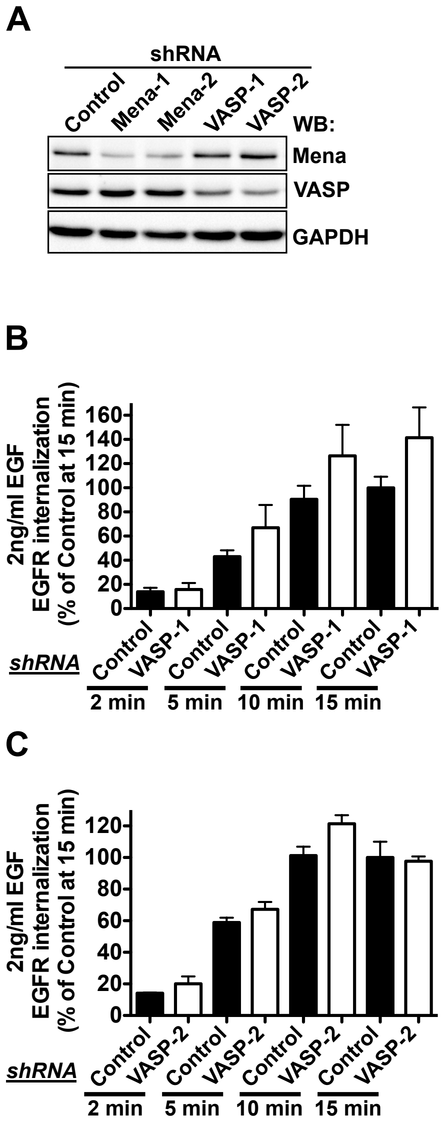

Supplemental Figure 5, Vehlow et al.

Figure S5: Mena but not VASP regulates CME of the EGFR

(A) Detection of endogenous Mena and VASP using specific antibodies in lysates of HeLa cells expressing two different Mena- or VASP-specific or control shRNA. GAPDH serves as loading control. **(B-C)** EGFR internalization in HeLa cells expressing two VASP-specific or control shRNA and treated with 2 ng/ml EGF for indicated times. Results are mean ± SEM of at least 3 independent experiments.

Movie S1: Lpd localizes to spots at the plasma membrane in addition to its known localization at the leading edge of cells.

Localization of Lpd-GFP was analysed in B16F1 cells by TIRF microscopy.

Movie S2: VASP localizes to CCPs.

Co-localization of GFP-VASP (shown in green) and mRFP-Clc (shown in magenta) at CCPs was analysed in HeLa cells by TIRF microscopy. The arrow highlights a CCP scission event (related to Figure S3A and S3B).

Movie S3: Lpd localizes to CCPs before scission.

Co-localization of Lpd-GFP (shown in green) and mRFP-Clc (shown in magenta) at CCPs was analysed in HeLa cells by TIRF microscopy. The arrow highlights a CCP scission event (related to Figure 4I and 4J).
